# Nonlinear Effect of Preexisting Cranial Adjacent Disc Degeneration on Cumulative 12-Year Revision Risk Following Lumbar Fusions

**DOI:** 10.1097/BRS.0000000000004949

**Published:** 2024-02-02

**Authors:** Leevi A. Toivonen, Heikki Mäntymäki, Lorin M. Benneker, Hannu Kautiainen, Arja Häkkinen, Marko H. Neva

**Affiliations:** a Department of Orthopedics and Traumatology, Tampere University Hospital and Tampere University, Tampere, Finland; b Department of Orthopedic, Sonnenhofspital, Bern, Switzerland; c Primary Health Care Unit, Kuopio University Hospital, Kuopio, Finland; d Folkhälsan Research Center, Helsinki, Finland; e Faculty of Sport and Health Sciences, University of Jyväskylä, Jyväskylä, Finland

**Keywords:** disc degeneration, combined imaging score, pfirrmann, lumbar fusion, lumbar spine fusion, adjacent segment disease, ASD, adjacent segment pathology, reoperation, revision surgery

## Abstract

**Study Design.:**

Retrospective analysis of prospectively collected data.

**Objective.:**

To evaluate how preexisting adjacent segment degeneration status impacts revision risk for adjacent segment disease (ASD) after lumbar fusions.

**Summary of Background Data.:**

ASD incurs late reoperations after lumbar fusion surgeries. ASD pathogenesis is multifactorial. Preexisting adjacent segment degeneration, measured by Pfirrmann, is suggested as one of the predisposing factors. We sought to find deeper insights into this association by using a more granular degeneration measure, the combined imaging score (CIS).

**Patients and Methods.:**

A total of 197 consecutive lumbar fusions for degenerative pathologies were enrolled in a prospective follow-up (median: 12 yr). Preoperative cranial adjacent segment degeneration status was determined using Pfirrmann and CIS, which utilize both radiographs and magnetic resonance imaging. On the basis of CIS, patients were trichotomized into tertiles (CIS <7, CIS 7–10, and CIS >10). The cumulative ASD revision risk was determined for each tertile. After adjusting for age, sex, body mass index, sacral fixation, and fusion length, hazard ratios (95% CI) for ASD revisions were determined for each Pfirrmann and CIS score.

**Results.:**

Patients in the intermediate CIS tertile had a cumulative ASD revision risk of 25.4% (17.0%–37.0%), while both milder degeneration (CIS <7) [13.2% (6.5%–25.8%)] and end-stage degeneration (CIS >10) [13.6% (7.0%–25.5%)] appeared to be protective against surgical ASD. Pfirrmann failed to show a significant association with ASD revision risk. Adjusted analysis of CIS suggested increased ASD revisions after CIS 7, which turned contrariwise after CIS 10.

**Conclusions.:**

The effect of preexisting adjacent segment degeneration on ASD reoperation risk is not linear. The risk appears to increase with advancing degeneration but diminishes with end-stage degeneration. Therefore, end-stage degenerative segments may be considered to be excluded from fusion constructs.

**Level of Evidence.:**

Therapeutic 3.

Adjacent segment disease (ASD) is the main culprit for late revision surgeries after lumbar fusions.^1,2^ It is characterized by progressive degenerative changes next to the fusion segment that have led to neural compression and/or segmental pain.^3^ In this paper, “ASD” refers to symptomatic ASD. ASD etiology is considered multifactorial, partially being an inherent part of progressive spinal degeneration while also being accelerated by altered loading next to the fusion segment.^4,5^ Studies have reported ASD occurrences higher after fusion for degenerative conditions compared with isthmic spondylolisthesis, where spinal degeneration status outside the fusion segment is likely less advanced.^6^

There are several ways to quantify disc degeneration. The Pfirrmann classification is by far the most used and simple enough for clinical use.^7^ It has, however, flooring and ceiling effects.^7,8^ The combined imaging score (CIS) using both magnetic resonance imaging (MRI) and radiographs has been shown to have more discriminatory power in grading degeneration.^9^ Several studies have reported associations between preoperative Pfirrmann-graded adjacent disc degeneration status and future ASD.^10–13^ The predictive potential of CIS-graded degeneration status on ASD risk has not been explored.

This study attempted to determine the effect of preexisting adjacent segment degeneration on cumulative ASD revision risk in the 12-year follow-up of elective lumbar fusions for degenerative indications. The preoperative juxtacranial adjacent disc degeneration status per Pfirrmann and CIS was compared between surgical ASD patients and those not being revised in a cumulative follow-up.

## PATIENTS AND METHODS

### Patients

We identified all elective lumbar fusions performed for spinal stenosis (with or without spondylolisthesis) between 2008 and 2012 at a tertiary academic center. Only surgeries on the lumbar or lumbosacral areas were included, irrespective of fusion length. Surgeries for tumor, fracture, or infection, or surgeries after prior fusion procedures, were excluded.

During the data collection period, our unit performed all spinal fusions in its catchment area. Therefore, the study population can be seen as a population-based sample of elective lumbar fusion surgeries. Clinical and surgical details were prospectively entered into a local spine database. Local Ethics Committee approval of Tampere University Hospital (R07108) was obtained before inception.

### Surgeries

All surgeries were open; posterior fusions were performed through a midline incision with pedicle screws and necessary decompressions inside the fusion construct. No decompressions were performed on the adjacent segment. Interbody spacers (posteriorly inserted) were used at the surgeon’s discretion.

### Radiologic Measurements

On the basis of some earlier studies^14,15^ and our experience suggesting cranial ASD to be more frequent than caudal ASD, we chose to study solely cranial adjacent segment fate to simplify analysis. From preoperative radiographs and MRIs, the cranial adjacent disc was graded by the following measures:

Schizas score^16^: a four-point scale to grade spinal stenosis (A, no or minor stenosis to D, extreme stenosis) on preoperative MRI.

Pfirrmann score^7^: a five-point scale to grade disc degeneration status (1, no or minor degeneration to 5, extreme degeneration). The classification is based on MRI findings. It is simple to use in everyday practice but is limited by the ceiling effect, failing to discriminate between advanced and severe degeneration.^8^

Combined imaging score (CIS)^9^: sum (0–17 points) of six subscores (three radiographic subscores and three MRI-based subscores, each ranging from 0 to 2 or three points). The classification was composed of parameters best correlating with degeneration assessed histologically and biochemically; the score shows a linear correlation to disc degeneration.^9^ Its subscores are depicted in Table 1. CIS 7 has been reported as the cutoff for severe degeneration.^9^ CIS gradings were performed by two authors (L.A.T. and H.M.), after which consensus was reached by choosing the intermediate or more severe option in the presence of disagreement. Raters achieved excellent agreement (ICC: 0.86, 95% CI: 0.82–0.89) after calibrating themselves to the instrument and each other.

**TABLE 1:** The Combined Imaging Score (CIS) to Grade Intervertebral Disc Degeneration is the Sum of Six Subscores^1^

Score	Radiographic parameters			Magnetic resonance imaging parameters		
	Height loss	Osteophytes	Intradiscal calcification	T2-signal intensity	Modic changes	Nucleus shape
0	0%–10%	Margins rounded	No calcifications	Normal	Normal	Round/oval
1	10%–20%	Margins pointed	Rim calcification	Intermediate loss	Type I	Extension into inner annulus
2	20%–30%	<2 mm	Intranuclear calcification	Marked loss	Type II	Extension into outer annulus
3	>30%	>2 mm	—	Absent signal	Type III	Extension beyond outer annulus

### Follow-Up

We identified all spinal reoperations until May 19, 2023, and collected dates for the first revision due to cranial ASD. Also, possible death dates were collected. Therefore, each patient’s follow-up endured over 10 years, or it ended at the patient’s first revision or death.

### Statistics

Summary statistics were described using mean and SD, median and interquartile range (IQR), or numbers as percentages. The linearity across the CIS tertiles was evaluated using the Cochran-Armitage test (categorical variables), the linear-by-linear test (ordered), and the analysis of variance (continuous) with an appropriate contrast (orthogonal). Crude cumulative adjacent segment disease (ASD) revision rates were analyzed by Kaplan-Meier methods; the linearity of the cumulative function across three ordered CIS level groups was analyzed by the log-rank test. A possible nonlinear relationship between CIS and the hazard of ASD revision was assessed by using the three-knot-restricted cubic spline Cox proportional hazards model. The model included age, sex, body mass index, sacral fixation, and fusion length as covariates. We used Harrell’s *C* to evaluate the predictive ability between CIS and Pfirrman scores in the different models, and 95% CIs were created using bias-corrected bootstrapping (5000 replications). Correlation coefficients were calculated by the Spearman method. Statistical analyses were performed using STATA software (version 18.0), StataCorp LP, College Station, TX.

## RESULTS

A total of 215 eligible fusions were identified, of which 18 lacked preoperative imaging. Therefore, 197 consecutive patients [77% women; mean age (SD): 66 (10) yr] constituted the study population. Demographical and clinical data are depicted in Table 2. The majority of patients were retired (72%), had spinal stenosis with degenerative spondylolisthesis (79%), and underwent short segment fusions (one or two levels) (66%).

**TABLE 2:** Demographical and Clinical Data of Participants Per the Combined Imaging Score (CIS) Tertiles

	Combined imaging score (CIS) tertiles			*P* for linearity
	≤6, N=54	7–10, N=81	>10, N=62	
Women, n (%)	45 (83)	65 (80)	42 (68)	0.043
Age, mean (SD)	59 (11)	69 (8)	68 (9)	<0.001
BMI, mean (SD)	27.8 (5.0)	28.6 (4.3)	29.4 (4.0)	0.050
Smoking, n (%)	6 (11)	3 (4)	1 (2)	0.024
Working, n (%)	21 (39)	18 (22)	16 (26)	0.13
Duration of spinal complaints, mean (SD)	9.2 (9.1)	14.7 (13.9)	15.8 (15.2)	0.007
VAS, mean (SD)				
Back pain	60 (26)	62 (29)	64 (22)	0.37
Leg pain	68 (22)	70 (24)	65 (23)	0.46
ODI, mean (SD)	47 (15)	46 (15)	43 (16)	0.24
Comorbidities, n (%)				
Cardiovascular	26 (50)	46 (65)	39 (67)	0.069
Rheumatoid	4 (8)	6 (8)	4 (7)	0.87
Psychiatric	3 (6)	2 (3)	0	0.066
Pulmonary	1 (2)	8 (11)	1 (2)	0.89
Diabetes	5 (10)	10 (14)	8 (14)	0.52
Neurological	2 (4)	1 (1)	1 (2)	0.46
Indication for surgery, n (%)				0.060
LSS with DS	45 (83)	67 (83)	43 (69)	
LSS without DS	9 (17)	14 (17)	19 (31)	
Future adjacent level, n (%)				<0.001
T12/L1	0	0	2 (3)	
L1/2	2 (4)	10 (12)	22 (35)	
L2/3	24 (44)	34 (42)	24 (39)	
L3/4	27 (50)	35 (43)	12 (19)	
L4/5	1 (2)	2 (2)	2 (3)	
Preoperative adjacent level degeneration status				
Pfirrman grade, n (%)				<0.001
I	0	0	0	
II	21 (39)	3 (4)	0	
III	28 (52)	27 (33)	4 (6)	
IV	5 (9)	51 (63)	44 (71)	
V	0	0	14 (23)	
Schizas score for stenosis				<0.001
A	54 (100)	64 (79)	35 (56)	
B	0	16 (20)	27 (44)	
C	0	1 (1)	0	
D	0	0	0	
Fusion length				<0.001
1	21 (39)	21 (26)	11 (18)	
2	27 (50)	36 (44)	14 (23)	
3	6 (11)	21 (26)	23 (37)	
4	0	3 (4)	13 (21)	
5	0	0	1 (2)	
Interbody device, n (%)	7 (13)	7 (9)	6 (10)	0.58

DS indicates degenerative spondylolisthesis; LSS, lumbar spinal stenosis; ODI, Oswestry disability index; VAS, visual analog scale.

**Figure 1:**
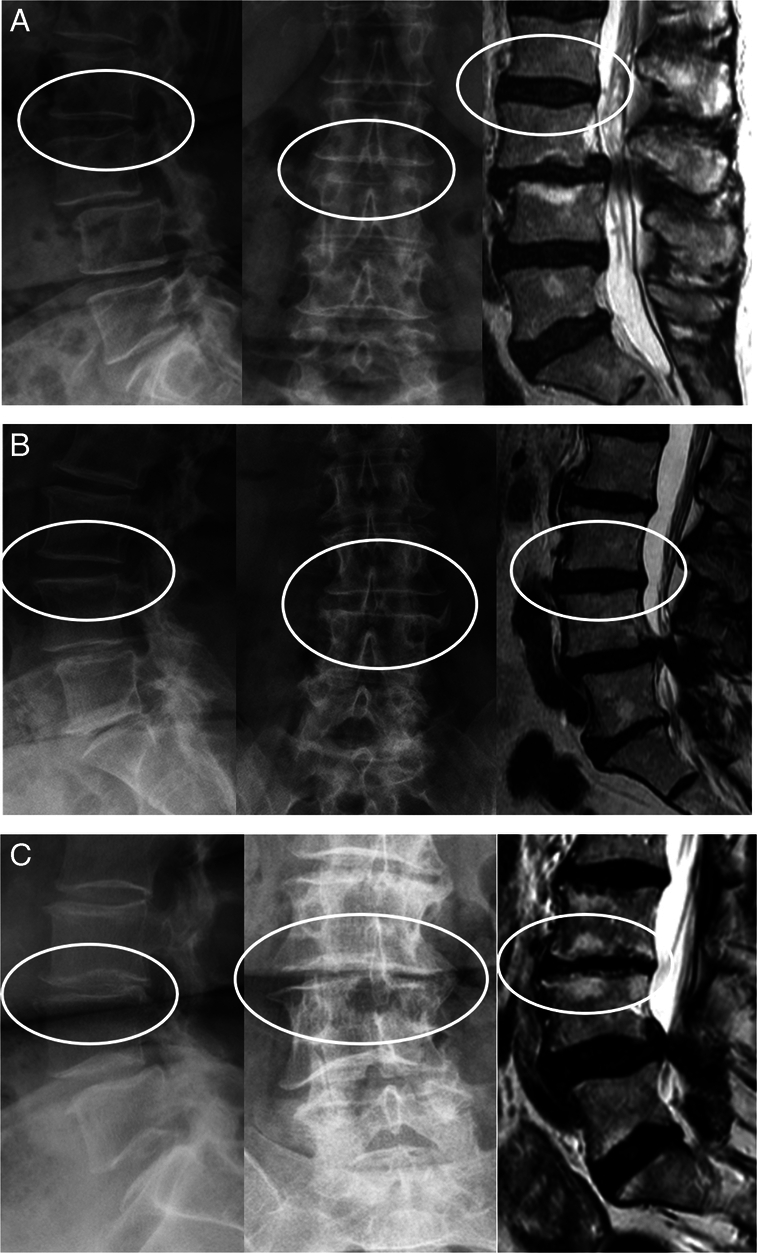
Illustrative cases of preoperative adjacent segment degeneration from each CIS tertile. A, Mild degeneration at L2-3 (CIS, 2+1+0+1+0+2=6) before L3-4 fusion. B, Mid-tertile degeneration at L3-4 (CIS, 1+3+0+2+0+2=8) before L4-S1 fusion. C, Severe degeneration at L3-4 (CIS, 3+1+0+3+2+3=12) before L4-5 fusion.

Over two-thirds of patients had severe degeneration at the cranial adjacent level before their index surgery (CIS 7 or greater). CIS >10 demarcates the most advanced degeneration tertile. Age and duration of spinal complaints were associated with the advancement of degeneration. More advanced degeneration was associated with longer fusions. Stenosis grade was, however, moderate at most at the future adjacent level before the index surgery. Illustrative examples of each CIS tertile are presented in Figure 1.

The median duration of follow-up was 12 years (IQR: 7–13 yr). At follow-up, 37 patients underwent revision surgery for cranial ASD. For consecutive CIS tertiles, these revision rates per 100 person years were: 1.3 (95% CI: 0.6–2.5) for the mild degeneration, 2.5 (1.5–4.0) for the middle, and 1.6 (0.7–2.8) for the most advanced degeneration tertile, respectively. There were five revisions for caudal ASD, but none of those patients later underwent surgery for cranial ASD.

**Figure 2:**
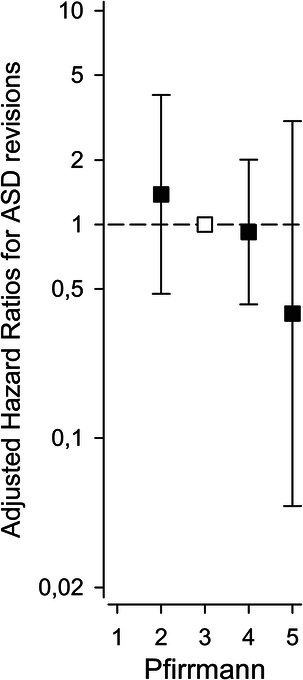
Adjusted hazard ratios (HR) (by age, sex, body mass index, sacral fixation, and fusion length) for adjacent segment disease (ASD) revisions per preoperative adjacent segment Pfirrmann grade. Reference was set to Pfirrmann 3.

Preoperative Pfirrmann grade of the future adjacent segment did not predict revisions for ASD (*P* for linearity 0.29) (Fig. 2). There was an insignificant trend toward fewer ASD revisions with end-stage degeneration (Pfirrmann 5).

**Figure 3:**
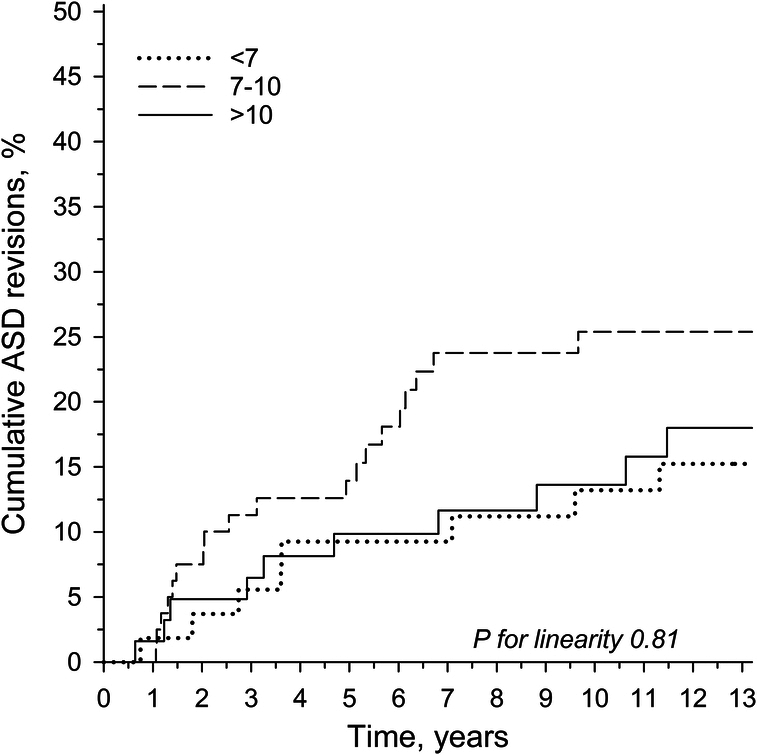
Crude cumulative adjacent segment disease (ASD) revision rates per preoperative adjacent segment disc degeneration status by the combined imaging score (CIS) tertiles.

**Figure 4:**
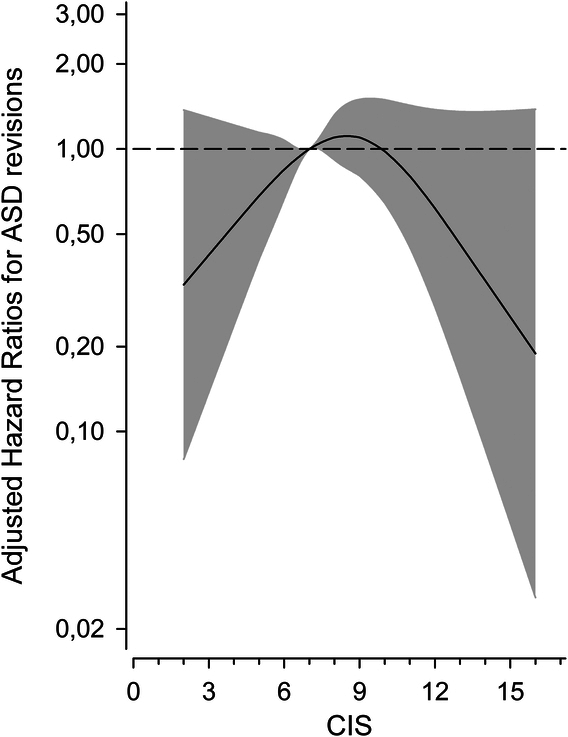
Adjusted (by age, sex, body mass index, sacral fixation, and fusion length) hazard ratios (HR) for adjacent segment disease (ASD) revisions per preoperative adjacent segment combined imaging score (CIS). The curve was derived from a three-knot restricted cubic splines Cox proportional hazards model. Reference was set to CIS 7.

When using a more granular degeneration measure, patients with severe adjacent segment degeneration (CIS 7–10) were more likely to end up in ASD revision surgery [crude 10-year risk, 25.4% (95% CI: 17.0%–37.0%)] than patients with less advanced degeneration [CIS <7: 10-year revision risk, 13.2% (6.5%–25.8%)] or most advanced degeneration [CIS >10: 10-year revision risk, 13.6% (7.0%–25.5%)]. The crude cumulative ASD revision rates are presented in Figure 3. A trend toward fewer ASD revisions with the mildest and most advanced adjacent segment degeneration status was also seen with adjusted (for age, sex, body mass index, sacral fixation, and fusion length) CIS scores (Fig. 4).

CIS and Pfirrmann performed equally in predicting ASD revisions [Harrell’s *C*: 0.60 (95% CI: 0.51–0.70), and 0.59 (0.49–0.68), respectively]. Overall correlation was strong between Pfirrmann and CIS [Spearman correlation coefficient: 0.75 (0.69–0.81)]. Of note, no single subscore of CIS was predictive of ASD revisions on its own.

## DISCUSSION

This study indicated that the connection between preexisting adjacent segment degeneration and subsequent surgical ASD is not linear. In fact, there appeared to be an inverted U-shape upturn in ASD revisions (the inflection point was CIS 7) with advanced juxtacranial degeneration status. End-stage degeneration, however, appeared to be protective against surgical ASD.

Our study failed to demonstrate any effect of the preexisting adjacent segment Pfirrmann grade on the risk of surgical ASD. Bagheri *et al.* ^17^ found only a limited effect of preoperative adjacent segment degeneration (Pfirrmann 3 as a cutoff) on symptomatic ASD (OR: 1.03). Wang *et al.* ^10^ demonstrated a stronger effect of preoperative adjacent level degeneration on surgical ASD (OR: 4.30), but they had no patients with Pfirrmann 4 or 5. Our patients, on the contrary, had preoperative adjacent-level degeneration of Pfirrmann ≥4 in 58% of cases. Therefore, the granularity of Pfirrmann was obviously insufficient here.

Benneker *et al.* ^9^ showed that integrating radiographic parameters into degeneration grading improves fidelity to discriminate morphologic and biochemical disc degeneration status. CIS combines the most influential parameters thereof. Its disadvantage is its complexity for routine clinical use.

When trichotomizing preoperative adjacent segment CIS scores, we found increased risk for surgical ASD with the intermediate tertile (CIS 7–10). CIS 7 has been reported as a threshold for severe degeneration.^9^ Both milder (CIS <7) and most severe degeneration (CIS >10) appeared to be protective against ASD. Despite advanced degeneration, our patients did not have severe stenosis at the adjacent level before the index procedure. That is reflective of the surgical strategy used and the generally advanced degenerative status of our patients. To our knowledge, no previous studies have investigated ASD occurrence based on preexisting degeneration graded by CIS.

There is a clear consensus that ASD pathogenesis is multifactorial.^2,5^ After controlling for plausible risk factors (age, sex, BMI, sacral fixation, and fusion length), the observed trend of peaking ASD revisions with intermediately severe preexisting degeneration remained insignificant. That underlines the complexity of the phenomenon. End-stage degeneration may lead to a collapsed, stable level that occasionally revolves even symptom-free, especially in the absence of stenosis.

### Strengths and Limitations

The overall good correlation between Pfirrmann and CIS grades supported our findings. Also, consistent notions of end-stage degeneration protecting against surgical ASD highlighted the discovery. We consider the use of Kaplan-Meier methods in analyzing cumulative phenomena like ASD optimal compared with existing studies that mostly dichotomize participants on the basis of accomplished follow-up. Revision surgery as an endpoint, however, is an express limitation of the present study, as there are no universal criteria for repeat surgeries. Radiologic or symptomatic ASD is reportedly only modestly associated with surgical ASD.^2,15^ Hence, ending up with repeat surgeries is still highly relevant for patients, surgeons, and stakeholders, making our setting valid. Also, our sample size is relatively small, which may reduce the power to reach statistical significance.

Elucidating the influence of a single factor on a complex, multifactorial phenomenon is cumbersome. Therefore, the prospects of (semiautomatic) artificial intelligence-based risk stratification tools for surgical contemplation appear alluring. The present findings suggest that including cranial end-stage degenerative, stable segments in fusion constructs may not be necessary. However, patients with moderately advanced degeneration above the fusion segment deserve counseling about ASD revision risk.

## CONCLUSIONS

The effect of preexisting adjacent segment degeneration on reoperation risk for ASD is not linear. The risk appears to increase with advancing degeneration but diminishes with end-stage degeneration. Therefore, end-stage degenerative segments may sometimes be excluded from contemplated fusion constructs.

Key Points

- We graded preoperative adjacent disc degeneration status by a combined imaging score.
- The effect of preoperative degeneration status on the cumulative 12-year reoperation risk for adjacent segment disease was not linear.
- Advanced degeneration increased the risk, but end-stage degeneration appeared to be protective against reoperations.

## References

[1] Kraemer P Fehlings MG Hashimoto R . A systematic review of definitions and classification systems of adjacent segment pathology. Spine (Phila Pa 1976). 2012;37(suppl):S31–S39. 22885835 10.1097/BRS.0b013e31826d7dd6

[2] Hashimoto K Aizawa T Kanno H . Adjacent segment degeneration after fusion spinal surgery-a systematic review. Int Orthop. 2019;43:987–993. 30470865 10.1007/s00264-018-4241-z

[3] Park P Garton HJ Gala VC . Adjacent segment disease after lumbar or lumbosacral fusion: review of the literature. Spine (Phila Pa 1976). 2004;29:1938–1944. 15534420 10.1097/01.brs.0000137069.88904.03

[4] Hilibrand AS Robbins M . Adjacent segment degeneration and adjacent segment disease: the consequences of spinal fusion? Spine J. 2004;4(suppl):190S–194S. 15541666 10.1016/j.spinee.2004.07.007

[5] Lau KKL Samartzis D To NSC . Demographic, surgical, and radiographic risk factors for symptomatic adjacent segment disease after lumbar fusion: a systematic review and meta-analysis. J Bone Joint Surg Am. 2021;103:1438–1450. 34166276 10.2106/JBJS.20.00408

[6] Toivonen LA Mäntymäki H Häkkinen A . Isthmic spondylolisthesis is associated with less revisions for adjacent segment disease after lumbar spine fusion than degenerative spinal conditions: a 10-year follow-up study. Spine (Phila Pa 1976). 2022;47:303–308. 35068470 10.1097/BRS.0000000000004242 PMC8772434

[7] Pfirrmann CW Metzdorf A Zanetti M . Magnetic resonance classification of lumbar intervertebral disc degeneration. Spine (Phila Pa 1976). 2001;26:1873–1878. 11568697 10.1097/00007632-200109010-00011

[8] Griffith JF Wang YX Antonio GE . Modified Pfirrmann grading system for lumbar intervertebral disc degeneration. Spine (Phila Pa 1976). 2007;32:E708–E712. 18007231 10.1097/BRS.0b013e31815a59a0

[9] Benneker LM Heini PF Anderson SE . Correlation of radiographic and MRI parameters to morphological and biochemical assessment of intervertebral disc degeneration. Eur Spine J. 2005;14:27–35. 15723249 10.1007/s00586-004-0759-4 PMC3476685

[10] Wang H Ma L Yang D . Incidence and risk factors of adjacent segment disease following posterior decompression and instrumented fusion for degenerative lumbar disorders. Medicine (Baltimore). 2017;96:e6032. 28151909 10.1097/MD.0000000000006032 PMC5293472

[11] Zhong ZM Deviren V Tay B . Adjacent segment disease after instrumented fusion for adult lumbar spondylolisthesis: Incidence and risk factors. Clin Neurol Neurosurg. 2017;156:29–34. 28288396 10.1016/j.clineuro.2017.02.020

[12] Liang J Dong Y Zhao H . Risk factors for predicting symptomatic adjacent segment degeneration requiring surgery in patients after posterior lumbar fusion. J Orthop Surg. 2014;9:97-97. 10.1186/s13018-014-0097-0 PMC4197214 25305779

[13] Hyun S-J Kim Y-B Hong H-J . Predictable risk factors for adjacent segment degeneration after lumbar fusion. J Korean Neurosurg Soc. 2007;41:88–94.

[14] Anandjiwala J Seo JY Ha KY . Adjacent segment degeneration after instrumented posterolateral lumbar fusion: a prospective cohort study with a minimum five-year follow-up. Eur Spine J. 2011;20:1951–1960. 21786038 10.1007/s00586-011-1917-0 PMC3207344

[15] Okuda S Nagamoto Y Matsumoto T . Adjacent segment disease after single segment posterior lumbar interbody fusion for degenerative spondylolisthesis: minimum 10 years follow-up. Spine (Phila Pa 1976). 2018;43:E1384–E1388. 29794583 10.1097/BRS.0000000000002710

[16] Schizas C Theumann N Burn A . Qualitative grading of severity of lumbar spinal stenosis based on the morphology of the dural sac on magnetic resonance images. Spine (Phila Pa 1976). 2010;35:1919–1924. 20671589 10.1097/BRS.0b013e3181d359bd

[17] Bagheri SR Alimohammadi E Zamani Froushani A . Adjacent segment disease after posterior lumbar instrumentation surgery for degenerative disease: incidence and risk factors. J Orthop Surg (Hong Kong). 2019;27:2309499019842378. 31046589 10.1177/2309499019842378

